# RETRACTED: Peritore et al. Ultramicronized Palmitoylethanolamide and Paracetamol, a New Association to Relieve Hyperalgesia and Pain in a Sciatic Nerve Injury Model in Rat. *Int. J. Mol. Sci.* 2020, *21*, 3509

**DOI:** 10.3390/ijms27083545

**Published:** 2026-04-16

**Authors:** Alessio Filippo Peritore, Rosalba Siracusa, Roberta Fusco, Enrico Gugliandolo, Ramona D’Amico, Marika Cordaro, Rosalia Crupi, Tiziana Genovese, Daniela Impellizzeri, Salvatore Cuzzocrea, Rosanna Di Paola

**Affiliations:** 1 Department of Chemical, Biological, Pharmaceutical and Environmental Science, University of Messina, 98165 Messina, Italy; aperitore@unime.it (A.F.P.); rsiracusa@unime.it (R.S.); rfusco@unime.it (R.F.); egugliandolo@unime.it (E.G.); rdamico@unime.it (R.D.); tgenovese@unime.it (T.G.); dimpellizzeri@unime.it (D.I.); dipaolar@unime.it (R.D.P.); 2 Department of Biomedical and Dental Sciences and Morphofunctional Imaging, University of Messina, 98165 Messina, Italy; cordarom@unime.it; 3 Department of Veterinary Science, University of Messina, 98165 Messina, Italy; rcrupi@unime.it; 4 Department of Pharmacological and Physiological Science, Saint Louis University School of Medicine, St. Louis, MO 63104, USA

The journal retracts the article titled “Ultramicronized Palmitoylethanolamide and Paracetamol, a New Association to Relieve Hyperalgesia and Pain in a Sciatic Nerve Injury Model in Rat” [1], cited above.

Following publication, concerns were brought to the attention of the Editorial Office regarding the integrity of images presented in this publication [1].

Adhering to our standard procedure, the Editorial Office and Editorial Board conducted an investigation that confirmed duplications between Figure 5a,b,d in [1] and sub-images in Figure 7 from a previous publication [2], presenting different experimental conditions. While the authors cooperated during the investigation, they were unable to satisfactorily explain the duplication, nor provide the suitably supporting material for Editorial Board evaluation. As a result, the Editorial Board has lost confidence in the reliability of the findings and has decided to retract this publication, as per MDPI’s retraction policy (https://www.mdpi.com/ethics#_bookmark30, accessed on 19 March 2026).

This retraction was approved by the Editor-in-Chief of the journal *IJMS*.

The authors do not agree with this decision.
